# Intrarater reliability of the Humac NORM isokinetic dynamometer for strength measurements of the knee and shoulder muscles

**DOI:** 10.1186/s13104-018-3128-9

**Published:** 2018-01-10

**Authors:** Bas Habets, J. Bart Staal, Marsha Tijssen, Robert van Cingel

**Affiliations:** 1 Sports Medical Center Papendal, Papendallaan 7, 6816 VD Arnhem, The Netherlands; 2 Department of Rehabilitation, Physical Therapy Science & Sports, University Medical Center Utrecht, Utrecht, The Netherlands; 3 Research Group Musculoskeletal Rehabilitation, HAN University of Applied Sciences, Nijmegen, The Netherlands; 4 Radboud University Medical Center, Research Institute for Health Sciences, IQ Healthcare, Nijmegen, The Netherlands

**Keywords:** Reliability, Technical report, Isokinetic dynamometry, Muscle strength, Knee, Shoulder

## Abstract

**Objective:**

To determine the intrarater reliability of the Humac NORM isokinetic dynamometer for concentric and eccentric strength tests of knee and shoulder muscles.

**Results:**

54 participants (50% female, average age 20.9 ± 3.1 years) performed concentric and eccentric strength measures of the knee extensors and flexors, and the shoulder internal and external rotators on two different Humac NORM isokinetic dynamometers, which were situated at two different centers. The knee extensors and flexors were tested concentrically at 60° and 180°/s, and eccentrically at 60° s. Concentric strength of the shoulder internal and external rotators, and eccentric strength of the external rotators were measured at 60° and 120°/s. We calculated intraclass correlation coefficients (ICCs), standard error of measurement, standard error of measurement expressed as a %, and the smallest detectable change to determine reliability and measurement error. ICCs for the knee tests ranged from 0.74 to 0.89, whereas ICC values for the shoulder tests ranged from 0.72 to 0.94. Measurement error was highest for the concentric test of the knee extensors and lowest for the concentric test of shoulder external rotators.

**Electronic supplementary material:**

The online version of this article (10.1186/s13104-018-3128-9) contains supplementary material, which is available to authorized users.

## Introduction

Isokinetic dynamometry is considered a valid instrument for assessing muscle strength, and it is often used as a reference standard for other strength assessments [1]. An isokinetic dynamometer allows to assess muscle function with an accommodating resistance, at a constant angular velocity, thereby enabling maximum force production throughout a prescribed range of motion (ROM) [2, 3].

In clinical practice, isokinetic dynamometry is often used to monitor progress during rehabilitation. For this purpose, good test–retest reliability of test results obtained with the same dynamometer is required, and this has been consistently demonstrated in various studies [4, 5]. However, isokinetic strength measurements are also widely used in a (multicenter) research context. For instance, in a recent study, we compared shoulder strength of female handball players to controls [6]. Both groups were measured by the same researcher, at different centers, using two different dynamometers of the same model. This was a choice of convenience based on availability of study participants. In this context, reliability of the test results obtained on two different dynamometers of the same model is required. To our knowledge, this has not previously been investigated. Therefore, the aim of this study was to investigate the intrarater reliability of two Humac NORM isokinetic devices in the assessment of concentric and eccentric strength measurements of the knee extensors and flexors, and the shoulder rotators.

## Main text

### Methods

#### Study design

This was a test–retest study, with an interval between the first and second test of 1–2 weeks. This was considered sufficient to minimize the influence of muscular fatigue, but also sufficiently short to ensure no actual change in strength [7]. The first test was performed at Papendal Sports Medical Center (Arnhem, The Netherlands), and the re-test was performed at HAN University of Applied Sciences (Nijmegen, The Netherlands).

All tests were conducted by two physiotherapy students, of which one performed the actual test, and the other checked all settings prior to commencement of the test. Both had limited experience in isokinetic dynamometry. They followed an intensive training program prior to the enrolment of participants to become familiar with the testing device and procedures.

#### Participants

The study sample consisted of healthy participants of 18–55 years of age. According to the COSMIN statement, we needed at least 50 participants for good methodological quality [8, 9]. No restrictions were made on (sports) activity level. Participants were excluded (1) if they had lower and/or upper extremity injuries that had limited their physical activity level in the past 6 months, (2) if they had surgery of the lower and/or upper extremity in the previous 12 months, (3) if they had a diagnosis interfering with a proper execution of the test, or (4) if they used medication that could influence their ability to deliver strength.

#### Procedures

All tests were performed on two Humac NORM isokinetic dynamometers (CSMi, Stoughton, MA), situated at two different centers. The software installed on the computers was similar (HUMAC 2009, v.9.7.1), and both dynamometers were calibrated prior to the start of the study according to the operating manual [10]. No gravity correction was used for all tests.

A fixed test sequence was chosen because of practical considerations. First, concentric and eccentric strength of the knee extensors/flexors was assessed at 60 and 180°/s, and subsequently concentric and eccentric tests for the shoulder rotators were performed at 60 and 120°/s (see Table 1). The first side to be tested was randomly determined by flipping a coin. During the re-test, participants started with the same side. Participants were asked not to perform any vigorous physical activity 48 h prior to their tests to minimize the influence of muscular fatigue.

**Table 1:** Content and sequence of the test procedure for isokinetic tests of the knee flexors/extensors and shoulder rotators

Knee flexors/extensors	Shoulder internal/external rotators
Concentric—concentric; 60°/s; 3 repetitions^a^	Concentric—concentric; 60°/s; 3 repetitions^a^
Concentric—concentric; 180°; 5 repetitions^a^	Concentric—concentric; 120°/s; 5 repetitions^a^
Concentric—eccentric extensors; 60°/s; 5 repetitions^a^	Concentric—eccentric external rotators; 60°/s; 5 repetitions^a^
Concentric—eccentric flexors; 60°/s; 5 repetitions^b^	Concentric—eccentric external rotators; 120°/s; 5 repetitions

^a^After each test, a 2 min rest interval was offered

^b^Between the knee and shoulder tests, a 5 min rest interval was offered, followed by warming-up

Prior to strength testing, participants performed 5 min of warming-up (70–80 revolutions per minute) either on a cycle ergometer (LifeFitness) for knee tests, or on a hand bike (LifeFitness) for shoulder tests. For familiarization with the Humac NORM and the test procedure, participants performed three submaximal and two maximal trial repetitions prior to each test [11]. A 30 s rest period was offered between trial repetitions and the test, and a 2 min rest interval after each test [12]. After finishing the tests for knee flexion/extension, participants rested 5 min, and subsequently they commenced their 5-min warming-up for the shoulder tests.

Before the start of the test, verbal instructions were given to push as hard and fast as possible through the full ROM. During the test, no verbal encouragement was made, but the computer screen was positioned so that participants could see real-time feedback of their effort.

#### Test for knee flexors/extensors

Participants were seated in an upright position, with the backrest at 85°. The rotational axis of the knee was placed in line with the dynamometer axis of rotation, and 0° was determined as 0° knee extension. The lever arm pad was secured just proximal to the medial malleolus, so that movement of the ankle was not constricted. Tests were performed in a predefined ROM of 90°–0°. To minimize compensatory trunk movements during testing, participants were secured using stabilizing straps, according to the manufacturer’s manual [10].

#### Test for shoulder rotators

Participants were placed supine with the shoulder in 90° of abduction, and the elbow flexed to 90°. The elbow was placed in the elbow stabilizer pad and then fixated by a velcro strap, so that the humeral shaft (i.e. shoulder axis of rotation) was in line with the axis of rotation of the dynamometer. Zero degrees of shoulder rotation was defined with the forearm in the neutral (vertical) position. The ROM of the test was between 50° internal rotation and 80° external rotation [13]. To minimize trunk movement, the trunk was secured with stabilizing straps and a velcro strap was placed over the iliac crest.

#### Statistical analysis

Statistical analyses were performed using SPSS 22.0 (α = 0.05). For all respective tests, the peak torque (PT, defined as the single highest point of the torque curve) of the best repetition (i.e. the repetition with the highest peak torque) was used for data analysis. To analyze systematic differences between test and re-test, a paired t test (two-tailed) was used. To correct for multiple testing, we applied a Bonferroni correction by dividing the p value by the amount of statistical comparisons.

For intrarater reliability, intraclass correlation coefficients (ICC_agreement_) (2,1) and 95% confidence intervals were calculated for all separate tests (i.e. knee extension and flexion, and shoulder internal rotation and external rotation). We used ICC_agreement_ to account for potential systematic differences between the first and second measurement [14]. An ICC of 0.40–0.59 was considered as ‘fair’, 0.60–0.74 as ‘good’, and > 0.75 as ‘excellent’ [15].

Measurement error was determined using standard error of measurement (SEM = standard deviation * (sqrt 1-ICC) [16], which is also presented as SEM% (SEM divided by the average of the first and second test). Additionally, the smallest detectable change at the 95% confidence level (SDC_95_ = 1.96 * SEM * sqrt 2) was calculated.

## Results

Fifty-four participants (50% female) were included (average age 20.9 ± 3.1 years, average height 175.2 ± 8.7 cm, and average weight 72.1 ± 12.3 kg). One participant could not complete the second knee test due to technical problems with the machine, whilst five participants (9%) did not perform the second shoulder test because of pain in the shoulder or wrist (n = 2) or a lack of time (n = 3). Data of the respective tests of these participants were therefore left out of the analysis.

### Knee flexors and extensors

No significant differences (p > 0.05) were found between the PTs of the test and re-test (Additional file 1), except for the concentric PT of the left knee extensors at 60°/s (p = 0.001), and for the eccentric PT of the left knee flexors at 60°/s (p < 0.001).

All tests showed good to excellent reliability, with ICC values ranging from 0.74 to 0.89 (Table 2). The SEM% ranged from 13 to 23.1%, with percentages for the concentric measures being slightly higher than for the respective eccentric measures.

**Table 2:** Intramachine reproducibility values for concentric and eccentric knee tests

	ICC (95% CI)	SEM (Nm)	SEM%	SDC (Nm)
KE concentric 60°/s				
Right	0.840 (0.728–0.907)	38.6	23.1	107.0
Left	0.762 (0.560–0.868)	30.7	19.8	85.1
KF concentric 60°/s				
Right	0.744 (0.596–0.843)	19.3	18.7	53.5
Left	0.804 (0.685–0.882)	17.3	17.0	48.0
KE concentric 180°/s				
Right	0.829 (0.723–0.897)	19.7	18.3	54.6
Left	0.861 (0.772–0.917)	17.7	17.1	49.1
KF concentric 180°/s				
Right	0.762 (0.617–0.856)	15.7	22.1	43.5
Left	0.826 (0.713–0.896)	13.5	19.4	37.4
KE eccentric 60°/s				
Right	0.849 (0.755–0.910)	27.7	14.8	76.8
Left	0.787 (0.659–0.871)	33.9	19.3	94.0
KF eccentric 60°/s				
Right	0.891 (0.817–0.936)	17.5	13.0	48.5
Left	0.840 (0.594–0.925)	20.9	16.0	57.9

*ICC* intraclass correlation coefficient, *CI* confidence interval, *SEM* standard error of measurement, *SDC* smallest detectable change, *KE* knee extensors, *KF* knee flexors

### Shoulder rotators

Analysis revealed a significant difference between the test and re-test for the eccentric external rotation at 60°/s (Additional file 2; p < 0.001).

Table 3 shows the reliability values of the respective shoulder tests, indicating good to excellent reliability for all tests (ICC ranging from 0.72 to 0.94). Measurement error (SEM%) ranged from 6.9 to 14.4%, and tended to be smaller for the external rotators than for the internal rotators.

**Table 3:** Intramachine reproducibility values for concentric and eccentric shoulder tests

	ICC_2,1_ (95% CI)	SEM (Nm)	SEM%	SDC (Nm)
SIR 60°/s				
Right	0.935 (0.888–0.963)	2.7	9.5	7.5
Left	0.924 (0.869–0.957)	2.7	9.8	7.5
SER concentric 60°/s				
Right	0.898 (0.811–0.944)	2.4	8.6	6.7
Left	0.934 (0.886–0.962)	1.9	6.9	5.3
SIR concentric 120°/s				
Right	0.878 (0.786–0.931)	3.7	14.4	10.3
Left	0.944 (0.897–0.969)	2.4	9.8	6.7
SER concentric 120°/s				
Right	0.813 (0.662–0.896)	3.0	12.1	8.3
Left	0.935 (0.882–0.964)	1.8	7.3	5.0
SER eccentric 60°/s				
Right	0.783 (0.385–0.906)	4.3	11.8	11.9
Left	0.905 (0.784–0.953)	3.1	8.6	8.6
SER eccentric 120°/s				
Right	0.722 (0.555–0.834)	4.6	13.0	12.8
Left	0.797 (0.665–0.881)	3.9	11.2	10.8

*ICC* intraclass correlation coefficient, *CI* confidence interval, *SEM* standard error of measurement, *SDC* smallest detectable change, *SIR* shoulder internal rotators, *SER* shoulder external rotators

## Discussion

To our knowledge, this was the first study to assess intrarater reliability for knee and shoulder tests performed on two different Humac NORM isokinetic dynamometers. We found good to excellent reliability, with ICC values ranging from 0.74 to 0.89 for knee tests and from 0.72 to 0.94 for shoulder tests. Nonetheless, our analysis revealed a significant difference between the test and re-tests, particularly for the eccentric tests. A possible explanation is that eccentric testing requires more extensive familiarization, as it may be more difficult for participants to produce maximal eccentric strength throughout the entire ROM [17]. Adding extra trial repetitions could have resulted in less variation in the values during the actual test [18], and would possibly have decreased the differences between the eccentric tests and re-tests.

Consistent with our findings, Impellizzeri et al. demonstrated ICC values of 0.90–0.98 for the concentric PT of the knee extensors and flexors obtained with a Cybex NORM dynamometer [19]. Several other studies have investigated intramachine reliability, and generally showed good reliability values, both for knee and shoulder testing [4, 5, 20, 21]. However, as these studies used different dynamometers, different test protocols, and gravity corrected data, comparison to our results should be done with caution.

Whilst relative reliability indicates how well subjects can be distinguished from each other, measurement error (SEM and SEM%) provides more clinically useful information. Our results showed SEM% values for the knee extensors ranging from 14.8 to 23.1%, and 13.0 to 22.1% for the knee flexors. A recent study that compared a Humac NORM to a Biodex dynamometer reported SEM values of 6.0–11.3 Nm for the knee extensors, and 3.7–6.8 Nm for the knee flexors, both at 60°/s (SEM% were not reported in this study) [22]. This is lower than the absolute SEM values for knee extensors and flexors found in our study, and may be explained by the fact that De Araujo Ribeiro Alvares et al. added an additional trial if the variation between the test trials (i.e. three repetitions) exceeded 10%. This is an important difference compared with our study, which could have decreased our measurement error values. Other differences were their study population (only males) and that their researchers were experienced in isokinetic dynamometry [22]. Impellizzeri et al. reported SEM% values for the knee extensors ranging from 4.0 to 6.8%, and 5.0 to 6.7% for the knee flexors [19], but the method to calculate SEM differed, hampering comparison to our results.

The SEM% values for the shoulder tests were considerably smaller than those found for the knee tests. The external rotators showed smaller measurement error than the internal rotators, although this difference was only small. We found one study that assessed reproducibility of internal and external rotation strength measures in the supine position on a Cybex NORM machine (60°/s) [23]. This study reported measurement error ranging from 10.5 to 11.8 for the internal rotators and 7.5–8.9 for the external rotators. However, comparison of those values to our results is difficult, as they used the coefficient of variation to express measurement error.

### Clinical relevance

This study shows good to excellent reliability values for concentric and eccentric strength measures of the knee extensors and flexors, and shoulder rotators, performed on two different Humac NORM isokinetic dynamometers. Nonetheless, measurement error was relatively high for the knee tests. Bearing the obtained results and particularly measurement error in mind, different devices of the same Humac NORM dynamometer can be used in a multicenter study.

## Limitations

- The current data apply to asymptomatic subjects and cannot automatically be translated to the clinical setting with a symptomatic population.
- The tests were performed by physiotherapy students with limited experience in isokinetic testing.

## Additional files

**Additional file 1.** Mean peak torque values ± standard deviations for tests and re-tests of the knee extensors and flexors.

**Additional file 2.** Mean peak torque values ± standard deviations for tests and re-tests of the shoulder internal and external rotators.

## Abbreviations

ROM: range of motion
PT: peak torque
ICC: intraclass correlation coefficient
SEM: standard error of measurement
SEM%: standard error of measurement expressed as a percentage
SDC: smallest detectable change

## References

[1] Stark T, Walker B, Phillips JK, Fejer R, Beck R (2011). Hand-held dynamometry correlation with the gold standard isokinetic dynamometry: a systematic review. PM R.

[2] Perrin DH (1993). Isokinetic exercise and assessment: leeds.

[3] Perrin DH (1994). Open chain isokinetic assessment and exercise of the knee. J Sport Rehabil.

[4] Orri JC, Darden GF (2008). Technical report: reliability and validity of the iSAM 9000 isokinetic dynamometer. J Strength Cond Res.

[5] Maffiuletti NA, Bizzini M, Desbrosses K, Babault N, Munzinger U (2007). Reliability of knee extension and flexion measurements using the Con-Trex isokinetic dynamometer. Clin Physiol Funct Imaging.

[6] van Cingel R, Habets B, Willemsen L, Staal B (2017). Shoulder dynamic control ratio and rotation range of motion in female junior elite handball players and controls. Clin J Sport Med.

[7] Lund H, Sondergaard K, Zachariassen T, Christensen R, Bulow P, Henriksen M, Bartels EM, Danneskiold-Samsoe B, Bliddal H (2005). Learning effect of isokinetic measurements in healthy subjects, and reliability and comparability of Biodex and Lido dynamometers. Clin Physiol Funct Imaging.

[8] De Vet HCW, Terwee CB, Mokkink LB, Knol DL (2011). Measurement in medicine.

[9] Terwee CB, Mokkink LB, Knol DL, Ostelo RW, Bouter LM, de Vet HC (2012). Rating the methodological quality in systematic reviews of studies on measurement properties: a scoring system for the COSMIN checklist. Qual Life Res.

[10] Computer Sports Medicine Inc (2003). Humac NORM testing and rehabilitation system—User’s guide, model 770.

[11] Van Cingel R, Kleinrensink GJ, Rooijens P, Uitterlinden E, Aufdemkampe G, Stoeckart R (2001). Learning effect in isokinetic testing of ankle invertors and evertors. Isokinet Exerc Sci.

[12] Almosnino S, Stevenson JM, Bardana DD, Diaconescu ED, Dvir Z (2012). Reproducibility of isokinetic knee eccentric and concentric strength indices in asymptomatic young adults. Phys Ther Sport.

[13] Van Cingel R, Kleinrensink GJ, Mulder P, De Bie R, Kuipers H (2007). Isokinetic strength values, conventional ratio and dynamic control ratio of shoulder rotator muscles in elite badminton players. Isokinet Exerc Sci.

[14] De Vet HC, Terwee CB, Knol DL, Bouter LM (2006). When to use agreement versus reliability measures. J Clin Epidemiol.

[15] Cicchetti DV (1994). Guidelines, criteria, and rules of thumb for evaluating normed and standardized assessment instruments in psychology. Psychol Assess.

[16] Denegar CR, Ball DW (1993). Assessing reliability and precision of measurement: an introduction to intraclass correlation and standard error of measurement. J Sport Rehabil.

[17] Harris-Love MO, Seamon BA, Gonzales TI, Hernandez HJ, Pennington D, Hoover BM (2017). Eccentric exercise program design: a periodization model for rehabilitation applications. Front Physiol.

[18] Kellis E, Baltzopoulos V (1995). Isokinetic eccentric exercise. Sports Med.

[19] Impellizzeri FM, Bizzini M, Rampinini E, Cereda F, Maffiuletti NA (2008). Reliability of isokinetic strength imbalance ratios measured using the Cybex NORM dynamometer. Clin Physiol Funct Imaging.

[20] Anderson VB, Bialocerkowski AE, Bennell KL (2006). Test-retest reliability of glenohumeral internal and external rotation strength in chronic rotator cuff pathology. Phys Ther Sport.

[21] Dauty M, Delbrouck C, Huguet D, Rousseau B, Potiron-Josse M, Dubois C (2003). Reproducibility of concentric and eccentric isokinetic strength of the shoulder rotators in normal subjects 40–55 years old. Isokinet Exerc Sci.

[22] de Araujo Ribeiro Alvares JB, Rodrigues R, de Azevedo Franke R, da Silva BG, Pinto RS, Vaz MA, Baroni BM (2015). Inter-machine reliability of the Biodex and Cybex isokinetic dynamometers for knee flexor/extensor isometric, concentric and eccentric tests. Phys Ther Sport.

[23] Forthomme B, Dvir Z, Crielaard JM, Croisier JL (2011). Isokinetic assessment of the shoulder rotators: a study of optimal test position. Clin Physiol Funct Imaging.

