# Supplementary material for: Intrarater reliability of the Humac NORM isokinetic dynamometer for strength measurements of the knee and shoulder muscles
Source: BMC Res Notes. 2018 Jan 10;11:15. doi: 10.1186/s13104-018-3128-9 (PMC5764011; doi:10.1186/s13104-018-3128-9)
Supplement: Supplementary file 1 — Additional file 1. Mean peak torque values ± standard deviations for tests and re-tests of the knee extensors and flexors. [file 13104_2018_3128_MOESM1_ESM.docx]

**Additional file 1.** Mean peak torque values (Nm) ± standard deviations for tests and re-tests of the knee extensors and flexors

|  | Test | Re-test | *p*-value |
| --- | --- | --- | --- |
| KE concentric 60°/sec  Right  Left | 172.2 ± 64.3  164.0 ± 62.9 | 161.1 ± 54.5  145.9 ± 51.1 | 0.015  0.001 |
| KF concentric 60°/sec  Right  Left | 103.7 ± 38.1  102.2 ± 39.1 | 103.0 ± 35.8  101.3 ± 30.3 | 0.863  0.777 |
| KE concentric 180°/sec  Right  Left | 106.3 ± 47.6  105.7 ± 47.6 | 108.7 ± 43.1  101.3 ± 42.3 | 0.526  0.170 |
| KF concentric 180°/sec  Right  Left | 68.0 ± 32.2  67.2 ± 32.3 | 74.2 ± 29.1  72.3 ± 28.4 | 0.034  0.035 |
| KE eccentric 60°/sec  Right  Left | 183.5 ± 71.2  177.6 ± 73.4 | 191.3 ± 80.8  174.5 ± 73.3 | 0.174  0.641 |
| KF eccentric 60°/sec  Right  Left | 137.9 ± 53.0  138.7 ± 52.3 | 132.1 ± 47.6  123.0 ± 44.7 | 0.075  <0.001 |

KE = knee extensors; KF = knee flexors
