# Supplementary material for: Intrarater reliability of the Humac NORM isokinetic dynamometer for strength measurements of the knee and shoulder muscles
Source: BMC Res Notes. 2018 Jan 10;11:15. doi: 10.1186/s13104-018-3128-9 (PMC5764011; doi:10.1186/s13104-018-3128-9)
Supplement: Supplementary file 2 — Additional file 2. Mean peak torque values ± standard deviations for tests and re-tests of the shoulder internal and external rotators. [file 13104_2018_3128_MOESM2_ESM.docx]

**Additional file 2.** Mean peak torque values (Nm) ± standard deviations for tests and re-tests of the shoulder internal and external rotators

|  | Test | Re-test | *p*-value |
| --- | --- | --- | --- |
| SIR 60°/sec  Right  Left | 28.9 ± 10.6  27.9 ± 9.9 | 28.2 ± 10.3  27.1 ± 9.5 | 0.174  0.131 |
| SER concentric 60°/sec  Right  Left | 28.4 ± 7.6  27.5 ± 7.4 | 27.1 ± 7.8  27.5 ± 7.6 | 0.010  0.918 |
| SIR concentric 120°/sec  Right  Left | 26.4 ± 10.6  25.1 ± 10.0 | 24.8 ± 9.4  24.1 ± 9.7 | 0.026  0.023 |
| SER concentric 120°/sec  Right  Left | 25.7 ± 7.0  24.2 ± 7.2 | 23.9 ± 7.5  25.1 ± 7.4 | 0.005  0.021 |
| SER eccentric 60°/sec  Right  Left | 38.5 ± 9.2  37.2 ± 10.1 | 34.5 ± 9.4  35.1 ± 9.7 | <0.001  <0.001 |
| SER eccentric 120°/sec  Right  Left | 36.1 ± 8.8  34.9 ± 8.6 | 34.7 ± 8.9  35.0 ± 8.7 | 0.159  0.897 |

SIR = shoulder internal rotators; SER = shoulder external rotators
